# Effect of Carbon-Based Modifications of Polydicyclopentadiene Resin on Tribological and Mechanical Properties

**DOI:** 10.3390/ma18204754

**Published:** 2025-10-16

**Authors:** Joanna Warycha, Janusz Kurowski, Jakub Smoleń, Krzysztof Stępień

**Affiliations:** 1 Department of Lightweight Engineering, Foundry and Automation, Wroclaw University of Science and Technology, 25 Smoluchowskiego Str., 50-370 Wroclaw, Poland; 2 IM Kompozyty, 8 Topolowa Str., Stanowice, 55-200 Oława, Poland; janusz@kompozyty.pl; 3 Department of Materials Technology, Silesian University of Technology, 8 Krasińskiego Str., Room 158A, 40-019 Katowice, Poland; jakub.smolen@polsl.pl (J.S.); krzysztof.stepien@polsl.pl (K.S.)

**Keywords:** self-lubricating bearings, PDCPD resin, flake/dusty graphite, carbon nanotubes, carbon fibers

## Abstract

Self-lubricating polymer composites based on polydicyclopentadiene (PDCPD) were reinforced with carbon nanomaterials to evaluate the effect of filler type and loading on their mechanical and tribological performance. Four carbon forms were introduced: carbon nanotubes (0.3 and 0.5 wt.%), carbon fibers (5 and 10 wt.%), flake graphite (5 and 10 wt.%) and dusty graphite (5 and 10 wt.%). Tensile tests showed that carbon fibers—and graphite-filled matrices reached ~50 MPa tensile strength, while the addition of carbon nanotubes resulted in a reduction in strength by half compared to the pure resin, indicating poor compatibility of carbon nanotubes with the matrix. The highest compressive strength, ~90 MPa, was obtained for PDCPD containing 5 wt.% carbon fibers. Tribological behavior was evaluated in a pin-on-disk configuration under dry sliding. All fillers lowered the coefficient of friction; the most pronounced, three-fold reduction was achieved with both graphite variants. The combined high load-bearing capacity and greatly reduced friction of the graphite and carbon fibers modified systems highlight their potential as self-lubricating bearing materials capable of replacing conventional metal or oil-lubricated components.

## 1. Introduction

With the continuous growth of industry—including the automotive [1], aerospace [2], and marine sectors, as well as wind [3] and hydropower [4] systems, electronics, and household appliances [5]—there is an increasing demand for advanced mechanical systems capable of maintaining reliable performance under harsh operating conditions, such as high temperatures, heavy loads, and high speeds [6,7,8,9].

It is well established that friction and material account for nearly 25% of global energy losses. In addition, the use of conventional lubricants involves significant maintenance costs and poses potential environmental risks. As a result, over the past two decades, increasing attention has been directed toward polymer-based self-lubricating materials, which can form a durable solid lubricant film during operation, thereby eliminating the need for external lubrication [10].

These polymeric systems, particularly self-lubricating composites, constitute a versatile class of engineering materials. They combine high mechanical strength, low density, corrosion resistance, and ease of fabrication, enabling continuous operation without external lubrication [11,12,13]. However, the mechanical demands placed on sliding bearings—especially in terms of load-carrying capacity and wear resistance—often exceed the intrinsic capabilities of pure polymers. To meet these requirements, polymer matrices are typically reinforced with fillers such as carbon fibers, carbon nanotubes, or graphite, which not only enhance mechanical performance but also serve as solid lubricants [14].

In self-lubricating polymer composites, the matrix material may be a thermoplastic such as polytetrafluoroethylene (PTFE) [15,16], polyamide (PA) [17], polyether ether ketone (PEEK) [18,19], polyethylene (PE) [20], or polyimide (PI) [21], as well as a chemosetting material such as epoxy resin [22]. Of particular interest is dicyclopentadiene (DCPD) resin, which exhibits a unique combination of thermoplastic and thermoset characteristics [23,24].

The polymer matrix determines the overall structure of the composite, distributes the applied loads, shields the filler from environmental influences, and governs the thermal and chemical stability of the material. The fillers, in turn, enhance the mechanical performance by increasing strength, reducing thermal expansion, improving wear resistance, and limiting crack propagation [25,26,27].

Polydicyclopentadiene (PDCPD) has gained increasing attention due to its rapid curing characteristics, exceptionally low viscosity (~10 cP), and low density (~1 g/cm^3^) [28]. Its mechanical performance is comparable to, or even surpasses, that of many conventional thermosetting polymers [29]. Moreover, PDCPD exhibits excellent impact resistance under high-strain-rate loading, a highly cross-linked molecular network, favorable vibration damping, chemical stability, and dimensional integrity—all of which are essential for demanding bearing applications [30,31,32].

Dicyclopentadiene (DCPD) is a colorless, camphor-like liquid obtained through the spontaneous Diels–Alder dimerization of cyclopentadiene. It represents a significant fraction of the C5 olefin stream derived from naphtha steam cracking. Only a portion of the total DCPD production—available in various purity grades—is recovered annually and utilized in applications such as tackifiers for adhesives and inks, as well as comonomers for polyester resins and EPDM rubbers [33,34,35,36,37,38,39,40].

Ring-opening metathesis polymerization (ROMP) of DCPD proceeds in two main stages. In the first step, the strained cyclopentene ring is opened by a catalyst, forming a linear structure at temperatures below 40 °C. In the second step, a strongly exothermic reaction promotes extensive cross-linking at temperatures above 80 °C [41].

Polydicyclopentadiene (PDCPD) is characterized by its extremely low monomer viscosity, which enables efficient infiltration into complex fiber preforms. As a monomeric resin, the infusion process is not hindered by polymer chain length [42]. Upon polymerization, PDCPD exhibits high impact strength, outstanding chemical resistance, and a rigid three-dimensional cross-linked network [43]. The literature reports emphasize that PDCPD attains mechanical strength comparable to, or even exceeding, that of epoxy resins, while offering a higher glass transition temperature (Tg) and superior vibration-damping capacity [41].

The use of PDCPD as a polymer matrix in carbon fiber–reinforced composites enables the fabrication of lightweight, high-strength structures with predictable impact performance. In recent years, thermoset polymers have been widely investigated for bearing applications; however, studies addressing the tribological performance of PDCPD are still limited. Its potential application in the development of self-lubricating bearings, therefore, offers promising opportunities for innovation in this field.

Graphite fillers—both flake and powdered—are widely employed in self-lubricating composites due to their layered crystal structure, high thermal conductivity, and excellent corrosion resistance [44,45,46]. Incorporating as little as 5–10 wt.% graphite into epoxy matrices has been shown to reduce the coefficient of friction by more than 30% [47]. Under extreme operating conditions, including elevated temperatures (100–350 °C) and high contact pressures (200–350 MPa), graphite can decrease wear depth by 60–80%, enhance mechanical strength, and promote the formation of protective tribofilms on the sliding surface [48].

Morstein and Dienwiebel [49] demonstrated that depositing graphite coatings with a thickness of 30–150 nm onto substrates of varying surface roughness extended service life by approximately 7.6 times, attributed to the formation of a stable carbon layer within the tribological interface.

Zhang et al. [50] reported that incorporating only 0.3 wt.% of silane-treated expanded graphite into PDCPD led to a 71.2% reduction in volumetric wear under dry “block-on-ring” test conditions. Scanning electron microscopy (SEM) confirmed the formation of a stable graphite transfer film on the counter surface. These findings indicate that even a small amount of graphite can significantly enhance wear resistance and extend the service life of sliding components.

Carbon fibers (CFs), whether continuous or short, are known to markedly enhance the tensile strength of PDCPD, achieving values exceeding 650 MPa when surface-treated to promote fiber–matrix adhesion [51]. Moreover, CF-reinforced PEEK bearings have demonstrated superior tribological performance compared to their glass fiber–reinforced counterparts [52]. In PDCPD composites, the incorporation of carbon fibers improves the strength-to-weight ratio and impact resistance compared with epoxy-based systems, although limited interfacial adhesion may still restrict efficient load transfer [32,51,52,53]. Despite their mechanical advantages, the friction and wear behavior of CF–PDCPD composites remains insufficiently explored.

Carbon nanotubes (CNTs) offer outstanding properties, like Young’s modulus ~1 TPa, thermal conductivity > 3000 W/m·K, low thermal expansion, intrinsic lubricity, and low density, making them excellent nano-reinforcements [14]. In a notable study, Jeong and Kessler added 0.4 wt.% functionalized CNTs to PDCPD, resulting in a ninefold increase in fracture energy and a shift to ductile failure [54]. Research on their mechanical, electrical, and tribological impacts in PDCPD remains limited [14,42,55].

Overall, most studies focus on a single filler type. Comparative studies involving CNTs, CFs, and graphite in the same PDCPD matrix are rare, and data linking filler content with combined mechanical and tribological performance are lacking, hampering the design of effective self-lubricating bearings.

This study addresses these gaps by examining four carbon filler morphologies—carbon fibers, carbon nanotubes, flake graphite, and dusty graphite —across 0.3–10 wt.% in PDCPD composites. We report tensile and compressive strengths, as well as friction performance in pin-on-disk tests. Our aim is to identify filler compositions that optimize load-bearing and friction reduction and to propose guidelines for developing maintenance-free, oil-free PDCPD-based bearing systems.

## 2. Materials and Methods

### 2.1. Materials

In this work, a matrix containing polydicyclopentadiene resin monomer DCPD (UL-TRENE 99-6 DICYCLOPENTADIENE—Cymetech Corporation, Calvert City, KY, USA), a ruthenium catalyst (AS2098-D1—Aperion Synthesis S.A., Wroclaw, Poland), and an accelerator (AS4011 C7—Aperion Synthesis S.A., Wroclaw, Poland) was used.

To improve the tribological and mechanical properties, carbon fillers selected from carbon fibers (ground carbon fiber powder)—38 μm, carbon nanotubes (multi-walled carbon nanotubes)—3–15 nm, flake graphite (Sinograf S.A., Toruń, Poland)—5 μm, and dusty graphite (Sinograf S.A., Toruń, Poland)—44 μm were added to the matrix.

### 2.2. Fabrication of Composite

The investigated composites were fabricated starting with the preparation of the polymer matrix. The components were measured in a quantity to obtain a volumetric ratio of 100 mL of dicyclopentadiene monomer to 0.5 mL of a ruthenium catalyst and 1 mL of an accelerator.

This formulation served as the base matrix for both the reference (unfilled) samples and those modified with carbon-based fillers.

The fillers used in the study included carbon fibers (added in amounts of 5 and 10 wt.%), carbon nanotubes (0.3 and 0.5 wt.%), and graphite in two morphological forms—flake graphite and dusty graphite, each added separately at 5 and 10 wt.%.

The prepared mixtures were subjected to mechanical stirring until a uniform dispersion was achieved. The homogenized materials were then poured into metal molds in the shape of dog-bone specimens with dimensions of 170 mm × 10 mm × 4 mm for tensile testing, as well as into beam-shaped molds with dimensions of 10 mm × 10 mm × 200 mm, from which samples were then cut for compressive strength and tribological testing.

The total time for mixing, homogenization, and casting did not exceed 20 min, as preliminary prepolymerization occurs beyond this period.

After reaching the pre-gel stage, the samples were removed from the molds and subsequently post-cured at 120–130 °C for 2–4 h to complete the polymerization process and ensure full curing of the PDCPD matrix.

### 2.3. Methods

#### 2.3.1. Mechanical Properties

**Figure 1:**
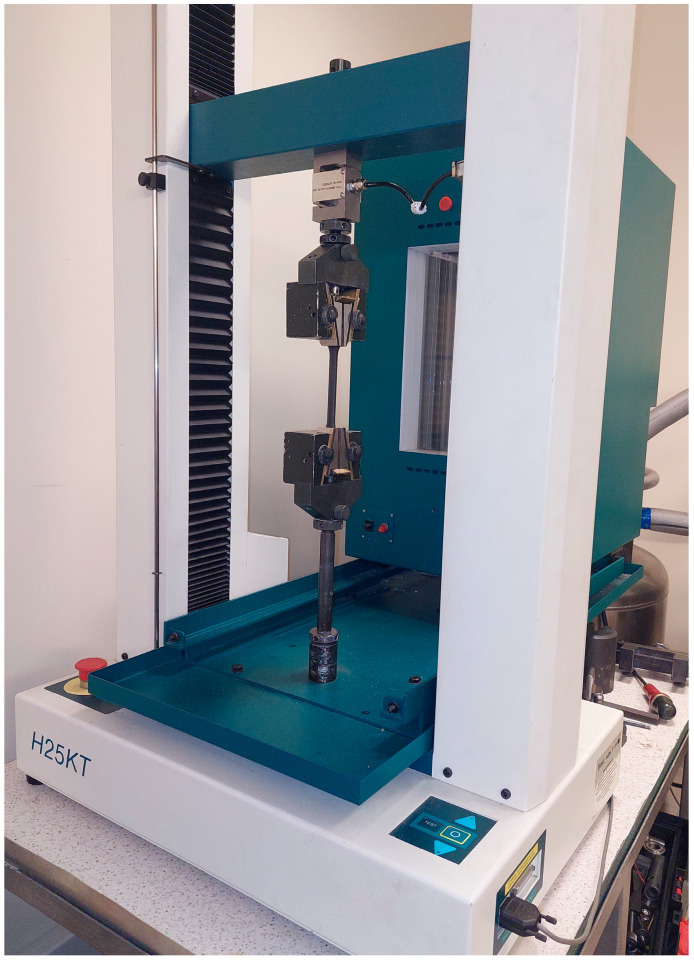
The figure of the research setup for compression and tensile tests.

Compression and tensile tests were performed using a Tinius Olsen H25KT (Tinus Olsen, KT, Philadelphia, PA, USA) testing machine operating at a crosshead speed of 10 mm/min. For tensile testing, dumbbell-shaped specimens with dimensions of 170 mm (±0.5 mm) × 10 mm (±0.2 mm) × 4 mm (±0.2 mm) were prepared, while compression tests were carried out on cube-shaped samples with a side length of 10 mm (±0.2 mm). Each test was conducted on a set of three specimens per filler type. The figure of the research setup is shown in Figure 1.

#### 2.3.2. Tribological Properties

**Figure 2:**
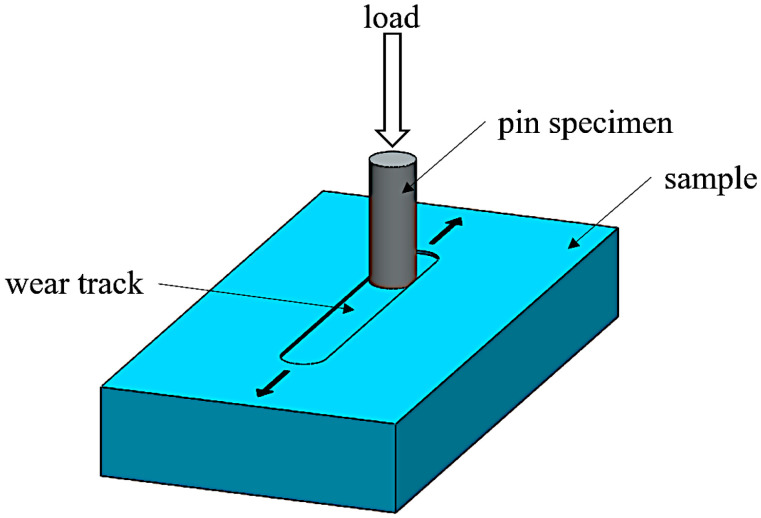
Schematic of the measurement setup for tribological tests using the pin-on-block method.

The tribological properties of the tested materials were determined using the pin-on-block tribological test, which allows for the determination of the dynamic coefficient of friction. The tests were conducted using a TM-01M tribotester. A reciprocating motion was used, with a total sliding distance of 200 m. The friction path was 12 mm, and the frequency was 4.17 Hz. The test lasted 2000 s, during which approximately 8333 cycles were performed. The counter-sample material was a steel pin (hardened carbon steel with a hardness of 60 ± 2 HRC, DIN 100Cr6) with a diameter of 6 mm and a flat end face shape. The sliding velocity was set at 0.1 m/s, and the pin pressure on the sample surface was 1 MPa. The tests were conducted at room temperature, approximately 23 °C. The diagram of the research setup is shown in Figure 2.

## 3. Results and Discussion

The specimens, after appropriate preparation, were tested for their mechanical properties, including tensile, compressive, as well as tribological evaluation. The results are summarized in Figure 3, Figure 4, Figure 5, Figure 6, Figure 7, Figure 8, Figure 9, Figure 10 and Figure 11 and Table 1 and Table 2.

### 3.1. Mechanical Properties

**Figure 3:**
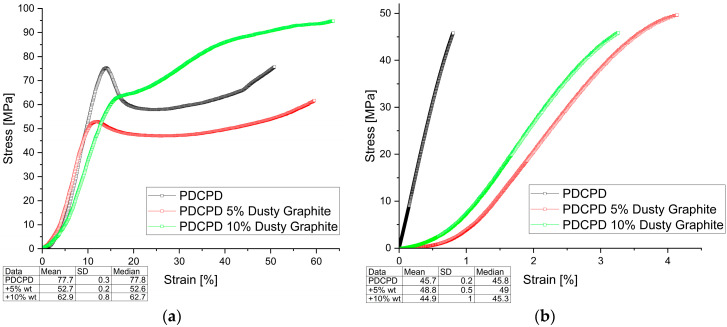
Mechanical behavior (**a**) compressive strength; (**b**) tensile strength for PDCPD resin + 5% and 10% dusty graphite.

**Figure 4:**
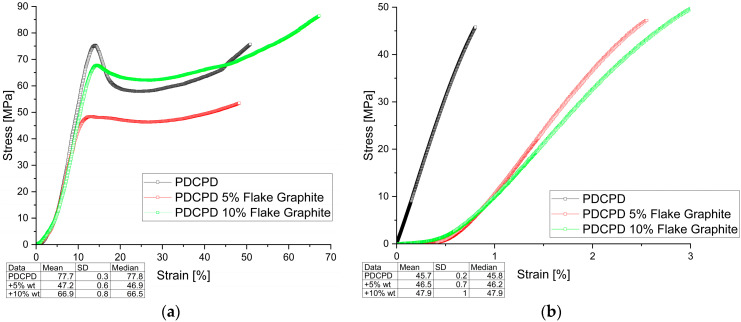
Mechanical behavior (**a**) compressive strength; (**b**) tensile strength for PDCPD resin + 5% and 10% flake graphite.

**Figure 5:**
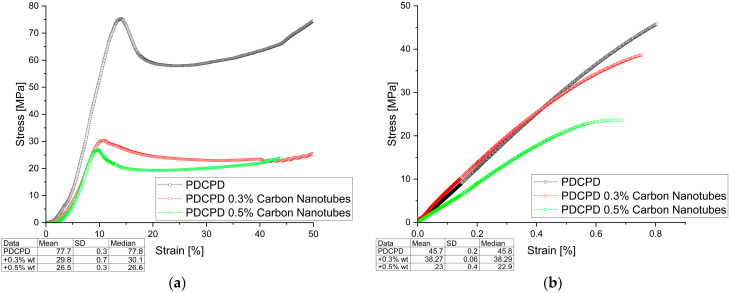
Mechanical behavior (**a**) compressive strength; (**b**) tensile strength for PDCPD resin + 0.3% and 0.5% carbon nanotubes.

**Figure 6:**
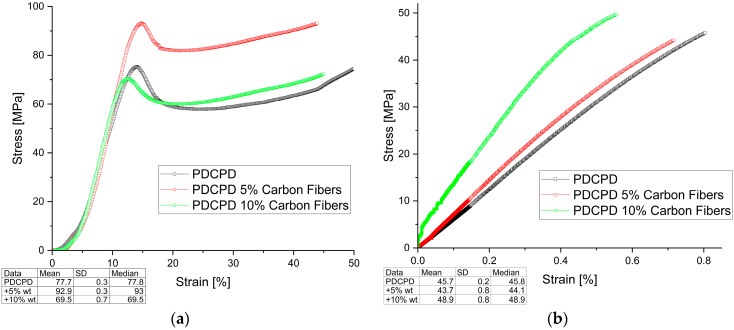
Mechanical behavior (**a**) compressive strength; (**b**) tensile strength for PDCPD resin + 5% and 10% carbon fibers.

The mechanical test results are shown in Figure 3, Figure 4, Figure 5 and Figure 6 (Compressive and Tensile strength) and summarized in Table 1, which presents the strength results for the best samples, converted to Stress [MPa].

Figure 3 illustrates the variation in compressive and tensile strength as a function of strain for PDCPD composites containing dusty graphite. Incorporation of 5 wt.% dusty graphite resulted in a slight increase in tensile strength, reaching 49.2 MPa. However, increasing the filler content to 10 wt.% led to a slight decrease in tensile strength, down to 45.8 MPa. A similar trend was observed for compressive strength, which was 52.9 MPa for the 5 wt.% addition and 63.9 MPa for the 10 wt.% addition of dusty graphite.

Figure 4 shows the results of compressive and tensile strength tests for PDCPD composites modified with flake graphite. As with powdered graphite, improved strength properties were observed for samples with 10 wt.% flake graphite. In the tensile strength tests for samples with 5 wt.% by weight and 10 wt.% by weight of flake graphite addition, a slight increase to 47.4 MPa and 48.9 MPa was noted, while in the compression tests, a decrease to 47.9 MPa and 67.9 MPa, respectively, compared to the unmodified matrix.

Figure 5 shows the changes in compressive and tensile strength of PDCPD composites modified with carbon nanotubes. The addition of 0.3 wt.% and 0.5 wt.% carbon nanotubes significantly reduced the compressive and tensile strengths. The compressive strengths were 30.4 MPa and 26.8 MPa, and the tensile strengths were 38.3 MPa and 23.5 MPa, respectively.

Figure 6 shows the variation in compressive and tensile strength of PDCPD composites with carbon fibers addition. The introduction of short carbon fibers significantly improved both compressive and tensile strength. Increasing the fiber content favors an increase in tensile strength—49.7 MPa for 10 wt.% carbon fiber content in the PDCPD matrix. Compressive strength increased by up to 20%, reaching 93.2 MPa for samples with 5 wt.% carbon fiber addition. The addition of 10 wt.% carbon fibers does not have a significant effect on strength, which may be due to inappropriate dispersion of the additive in the PDCPD matrix.

**Table 1:** Mechanical properties.

Samples	Compressive Strength [MPa]	Standard Deviation	Strain [%]	Tensile Strength [MPa]	Standard Deviation	Strain [%]
PDCPD	78.0	0.3	14.0	45.9	0.2	0.8
PDCPD + 5% Dusty Graphite	52.9	0.2	11.4	49.2	0.5	4.1
PDCPD + 10% Dusty Graphite	63.9	0.8	15.0	45.8	1.0	3.2
PDCPD + 5% Flake Graphite	47.9	0.6	11.9	47.4	0.7	2.5
PDCPD + 10% Flake Graphite	67.9	0.8	14.1	48.9	1.0	2.9
PDCPD + 0.3% Carbon Nanotubes	30.4	0.7	10.6	38.3	0.06	0.7
PDCPD + 0.5% Carbon Nanotubes	26.8	0.3	9.3	23.5	0.4	0.6
PDCPD + 5% Carbon Fibers	93.2	0.3	14.0	44.2	0.8	0.7
PDCPD + 10% Carbon Fibers	70.2	0.7	12.1	49.7	0.8	0.5

In tensile strength tests, all composites reinforced with carbon fibers or graphite (both flake and dusty) exhibited comparable or higher tensile strength compared to the unmodified PDCPD resin, which reached 45.5 MPa. The greatest improvement was recorded for the composite containing 10 wt.% carbon fibers, achieving a maximum tensile strength of 49.7 MPa.

The strain values obtained during the tensile tests ranged from 0.5% to 4.1%. Samples modified with carbon fibers and carbon nanotubes showed strain behavior comparable to that of the neat resin (0.8%), whereas those containing flake graphite reached strain values of up to 2.9%, and dusty graphite up to 4.1%. Interestingly, these variations in strain did not significantly influence the tensile strength results.

Compressive strength tests confirmed the reinforcing effect of carbon fiber addition. The composite containing 5 wt.% carbon fibers achieved a compressive strength of approximately 93.2 MPa, compared to 78.0 MPa for the unreinforced matrix. However, the incorporation of graphite—regardless of its form—resulted in a slight decrease in compressive strength.

The strain values obtained during the compression tests ranged from 9% to 15%. Samples modified with carbon fibers and carbon nanotubes exhibited slightly lower strain values than the unmodified resin (14%), whereas those containing flake or dusty graphite showed comparable strain values, up to 15%.

A significant deterioration in both tensile and compressive strength was observed in the samples modified with carbon nanotubes. In particular, the samples containing 0.5 wt.% CNTs exhibited a reduction of nearly 50% in tensile strength and almost 70% in compressive strength compared to the unreinforced resin. This degradation may be attributed to the unusually soft and rubbery texture of the cured samples, which suggests incomplete polymerization. A likely explanation is that the nanotubes adsorbed a significant portion of the catalyst, thereby reducing its effectiveness and hindering proper resin cross-linking.

### 3.2. Tribological Properties

**Figure 7:**
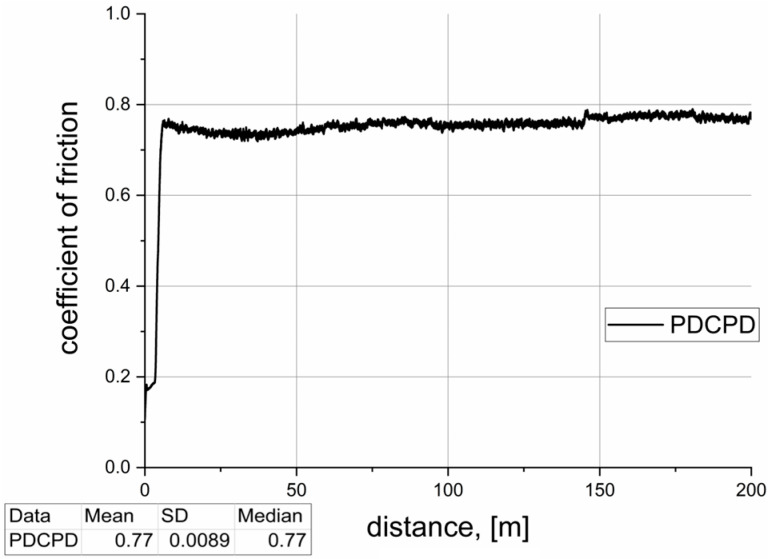
Tribological properties—PDCPD resin.

**Figure 8:**
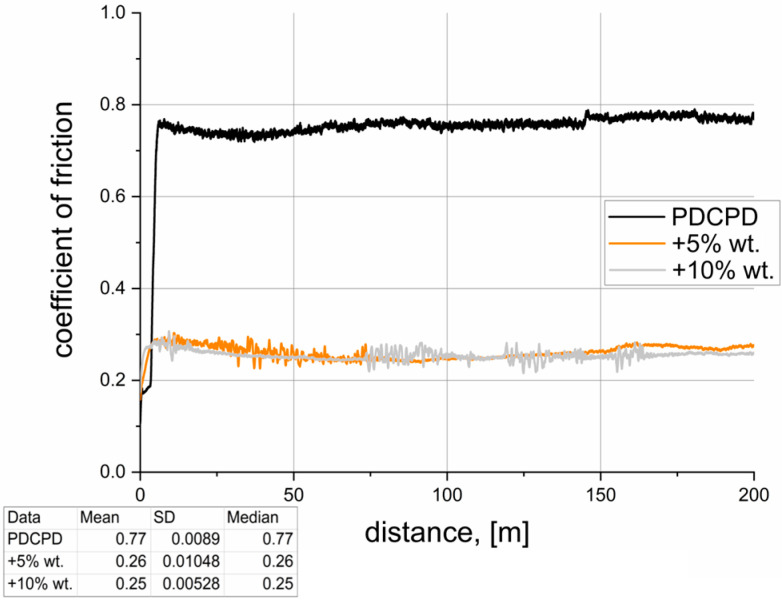
Tribological properties—PDCPD resin + dusty graphite.

**Figure 9:**
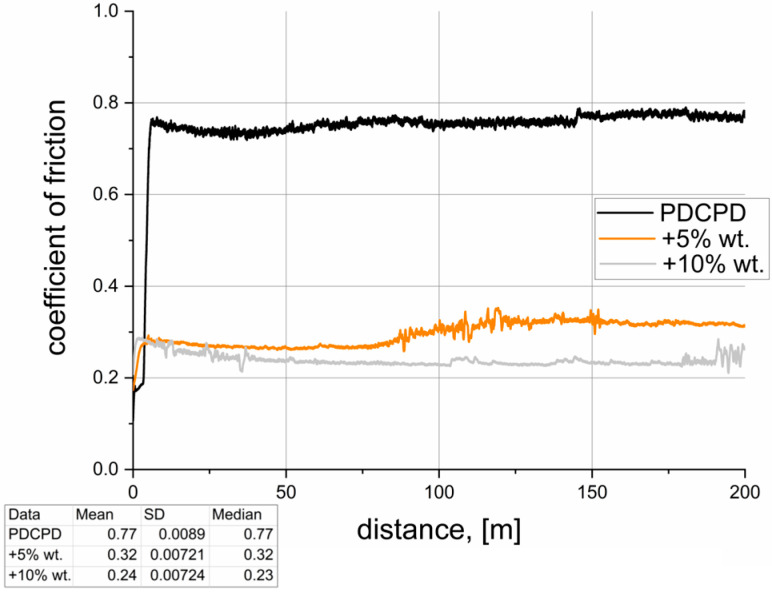
Tribological properties—PDCPD resin + flake graphite.

**Figure 10:**
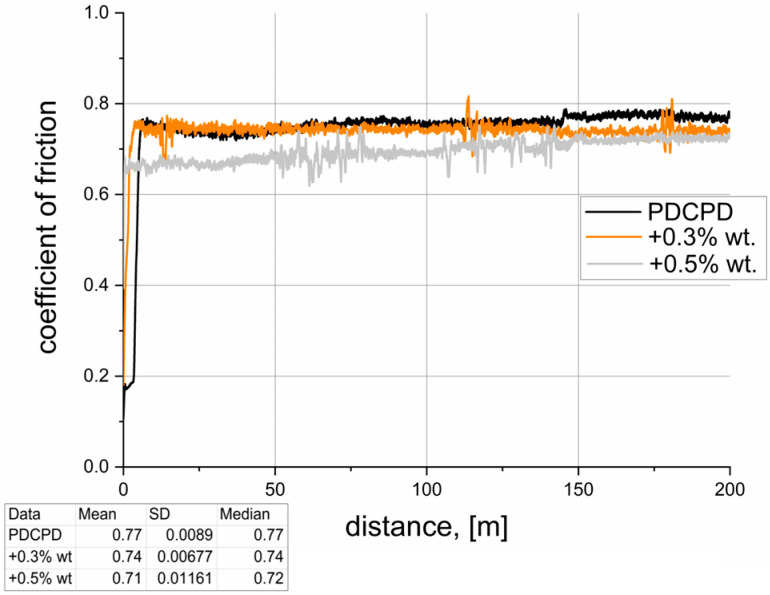
Tribological properties—PDCPD resin + carbon nanotubes.

**Figure 11:**
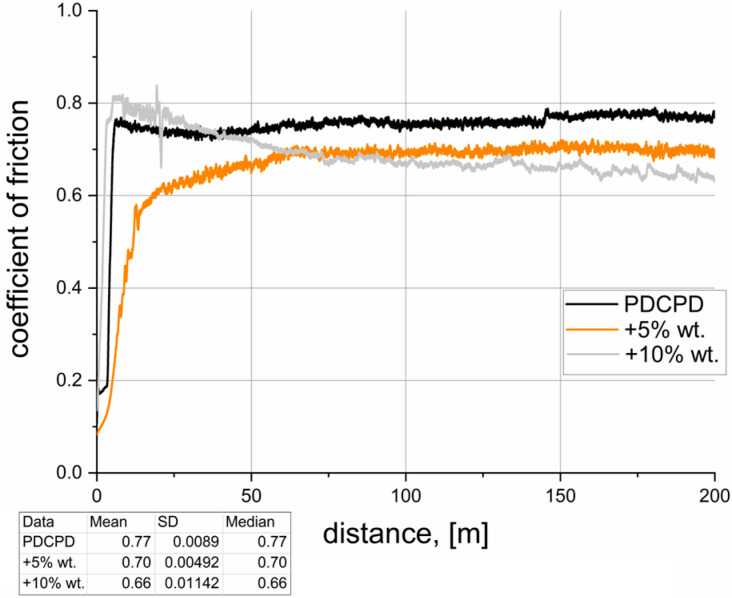
Tribological properties—PDCPD resin + carbon fibers.

Table 2 summarizes the results of tribological tests conducted for the analyzed materials. The dynamic coefficient of friction versus sliding distance, recorded during pin-on-block tests over a distance of 200 m, is illustrated in Figure 7, Figure 8, Figure 9, Figure 10 and Figure 11. The wear test was carried out on one sample each. The frequency of data acquisition was 25 Hz. In order to reduce noise in the measurements, a 255-point moving average filter was applied to smooth the curve. The coefficients of friction were calculated from the steady-state region of the curve. The steady-state region for the calculation for all samples was assumed as 100–200 m.

**Table 2:** Tribological properties.

Samples	Coefficient of Friction (a.u.)	Standard Deviation	Mass Loss [g]
PDCPD	0.77	0.00890	0.0005
PDCPD + 5% Dusty Graphite	0.26	0.01048	0.0001
PDCPD + 10% Dusty Graphite	0.25	0.00528	0.0001
PDCPD + 5% Flake Graphite	0.32	0.00721	0.0001
PDCPD + 10% Flake Graphite	0.24	0.00724	0.0002
PDCPD + 0.3% Carbon Nanotubes	0.74	0.00677	0.0010
PDCPD + 0.5% Carbon Nanotubes	0.71	0.01161	0.0009
PDCPD + 5% Carbon Fibers	0.70	0. 00492	0.0007
PDCPD + 10% Carbon Fibers	0.66	0. 01142	0.0006

Figure 7 presents the variation in the coefficient of friction under technically dry sliding conditions over a distance of 200 m. After approximately 10 m, the coefficient stabilizes at around 0.77 ± 0.0089. Such a relatively high value, observed in contact with steel, suggests that the material—without modification—is not appropriate for use in sliding applications.

Figure 8 presents the variation in the coefficient of friction as a function of sliding distance for PDCPD composites with the addition of dusty graphite. The incorporation of 5 wt.% and 10 wt.% graphite resulted in a significant reduction in the friction coefficient to approximately 0.25–0.26, representing a decrease of more than 67%. The difference between the 5 wt.% and 10 wt.% graphite additions is not substantial, and both concentrations effectively promote the formation of a solid lubricating layer and lead to a pronounced reduction in friction.

Figure 9 presents the tribological test results for PDCPD composites modified with flake graphite. Similarly to the case of dusty graphite, a significant reduction in the coefficient of friction is observed. However, notable differences occur between the composites containing 5 wt.% and 10 wt.% of flake graphite. A higher graphite content ensures a more effective reduction in the coefficient of friction to approximately 0.24 (a decrease of around 70%), whereas the 5 wt.% addition reduces it to about 0.32 (a decrease of approximately 60%). The addition of 5 wt.% flake graphite appears insufficient to maintain effective solid lubrication on the surface; after approximately 50 m of sliding, once the solid lubricant is depleted, an increase in the coefficient of friction is observed, which may be detrimental to further wear resistance. Samples filled with powdered and flake graphite exhibit typical tribofilm formation mechanisms similar to those observed in other polymer composites with such additives, as described in numerous literature publications. For example, Yingshuang Shang et al., in their publication “The effect of micron-graphite particle size on the mechanical and tribological properties of PEEK composites,” demonstrated tribofilm formation visible in SEM images [47,56].

Figure 10 presents the changes in the coefficient of friction for PDCPD composites modified with carbon nanotubes. The addition of 0.3 wt.% and 0.5 wt.% of carbon nano-tubes does not result in a statistically significant change in the coefficient of friction (reduction below 10%). However, a trend can be observed where increasing the nanotube content leads to a proportional decrease in the coefficient of friction.

Figure 11 shows the variation in the coefficient of friction as a function of sliding distance for PDCPD composites with the addition of carbon fibers. The incorporation of 5 wt.% short carbon fibers do not lead to a significant change in the coefficient of friction compared to unmodified PDCPD. An increasing fiber content promotes a reduction in friction, with a 10 wt.% addition lowering the coefficient to approximately 0.66 (a reduction of about 12%). The 10 wt.% fiber-reinforced composites require an initial run-in period to form a solid lubricant layer on the surface. Stabilization of the coefficient of friction in this composite is observed after approximately 70–100 m of sliding.

### 3.3. Discussion

The results of the tribological tests clearly indicate that the incorporation of carbon fillers into PDCPD resin-based composites significantly reduces the coefficient of friction compared to the reference material.

These results confirm the effectiveness of resin modification to obtain self-lubricating materials. Similar results were obtained by J. Myalski et al. in their article investigating the effect of carbon additives in the form of glassy carbon, biocarbon, and graphene oxide on PA6 polyamide. The authors linked the reduction in the coefficient of friction to an increase in the crystallinity of the composite compared to neat polyamide [57]. The effect of graphite additive on reducing the coefficient of friction is also confirmed by an article by J. Smoleń et al., in which the addition of 10–30% graphite to an epoxy matrix resulted in a 40–45% reduction in the coefficient of friction [58]. Of all the additives analyzed—carbon nanotubes, carbon fibers, flake graphite, and dusty graphite—the best tribological properties were demonstrated by both flake and dusty graphite, achieving up to a three-fold reduction in the coefficient of friction compared to the unmodified PDCPD matrix. This significant improvement in tribological properties may be due to the specific layered structure of graphite, which promotes easy shearing between atomic planes, leading to the formation of a stable lubricant film on the contact surface.

Although other carbon additives also contributed to reducing the coefficient of friction, their effectiveness was comparatively lower. Carbon fibers, while beneficial for improving mechanical properties, exhibited only a moderate influence on tribological performance. In the case of carbon nanotubes, the lubricating effect was less pronounced, likely due to their low concentration and the potential challenges associated with proper resin cross-linking.

Carbon nanotubes can adsorb catalysts, block reactive functional groups, or form agglomerates that hinder the effective initiation and progression of the cross-linking reaction. The lack of functionalization and the formation of excessively thick dispersions increase the viscosity of the mixture, restricting molecular mobility, creating a physical barrier to network formation, and ultimately slowing down the curing process [59,60,61].

## 4. Conclusions

The application of self-lubricating materials in sliding bearings, based on PDCPD resin composites with carbon additives—particularly those containing graphite, which exhibited the lowest coefficient of friction (0.24) and a tensile strength of 50 MPa at approximately 3% strain—could significantly enhance device durability, reduce maintenance costs, and extend maintenance intervals. Moreover, the use of lubrication-free bearings would help maintain the cleanliness of both the equipment and the enclosed environments in which they operate.

By exploiting the mechanical and tribological properties of the developed composites, it is possible to design not only individual components, such as self-lubricating bearings, but also entire assemblies featuring locally optimized properties—combining high wear resistance with excellent load-bearing capacity, as required in engine housings or structural components.

Additionally, considering the other properties of PDCPD resin, such as physico-chemical properties, low weight, low water absorption, and corrosion resistance, the application range of such components is expanding, for example, in aircraft and equipment operating in harsh environments, such as salty marine environments.

The obtained data on mechanical and tribological properties can provide a basis for further design of PDCPD composites with optimized wear resistance, intended for use in components operating under dry friction conditions.

## Data Availability

The original contributions presented in this study are included in the article. Further inquiries can be directed to the corresponding author.
